# Supplementary material for: Palaeoproteomic Deconvolution of Physical and Genetic Collagen Mixtures
Source: Adv Sci (Weinh). 2026 Sep 16:e77807. Online ahead of print. doi: 10.1002/advs.77807 (PMC13583091; doi:10.1002/advs.77807)
Supplement: Supplementary file 1 [file ADVS-9999-e77807-s005.pdf]

Supplementary materials for

Palaeoproteomics deconvolution of physical and genetic collagen mixtures

*Ian Engels[1], Tristan Dedrie[2], Synnøve K. Mo[3], Simon Van de Vyver[4], Thijs R. A. Vandenbroucke[3], Kévin Di Modíca[5], Jan Decher[6], Alice Toso[7], Dieter Deforce[1], Simon Daled[1]†, Alexandra Burnett[1,2]†, Grégory Abrams[2,5]†, Maarten Dhaenens[1]†**

[1]Laboratory of Pharmaceutical Biotechnology, ProGenTomics, Ghent University, Ghent 9000, Belgium.

[2]ArcheOs Laboratory for Biological Anthropology, Faculty of Arts and Philosophy, Ghent University, Ghent 9000, Belgium.

[3]Department of Geology, Ghent University, Ghent 9000, Belgium.

[4]Department of Mathematics, Statistics and Computer Science, Faculty of Sciences, Ghent University, 9000 Ghent, Belgium.

[5]Scladina Cave Archaeological Centre, Espace muséal d'Andenne, Andenne 5300, Belgium.

[6]Leibniz-Institut zur Analyse des Biodiversitätswandels (LIB), Museum Koenig Bonn, Adenauerallee 127, 53113, Bonn, Germany.

[7]Bonn Center for ArchaeoSciences, Institut für Archäologie und Kulturanthropologie, Rheinische Friedrich-Wilhelms-Universität, Bonn, Germany.

* Corresponding author e-mail: maarten.dhaenens@ugent.be

† These authors jointly supervised this work.

Funding: This research was funded by a grant from the Special Research Fund (BOF) of Ghent University with funding code BOF.GOA.2022.0002.003

Keywords: Palaeoproteomics, Bioinformatics, Palaeontology, collagen, Bioarchaeology

This file includes:

Supplementary Figures: Figure S1 to S3

Supplementary Formulae

References

Other Supplementary Marerials for this manuscript include the following:

Data S1 (ZIP): Interactive HTML files of figure 2

Data S2 (ZIP): Interactive HTML files of figure 3

Data S3 (ZIP): MEGA11 alignment files of Hyaenidae measured sequences

Data S4 (ZIP): Interactive HTML files of figure 5

Data S5 (ZIP): Excel document containing the results and sample metadata of all performed analyses.

Supplementary figures

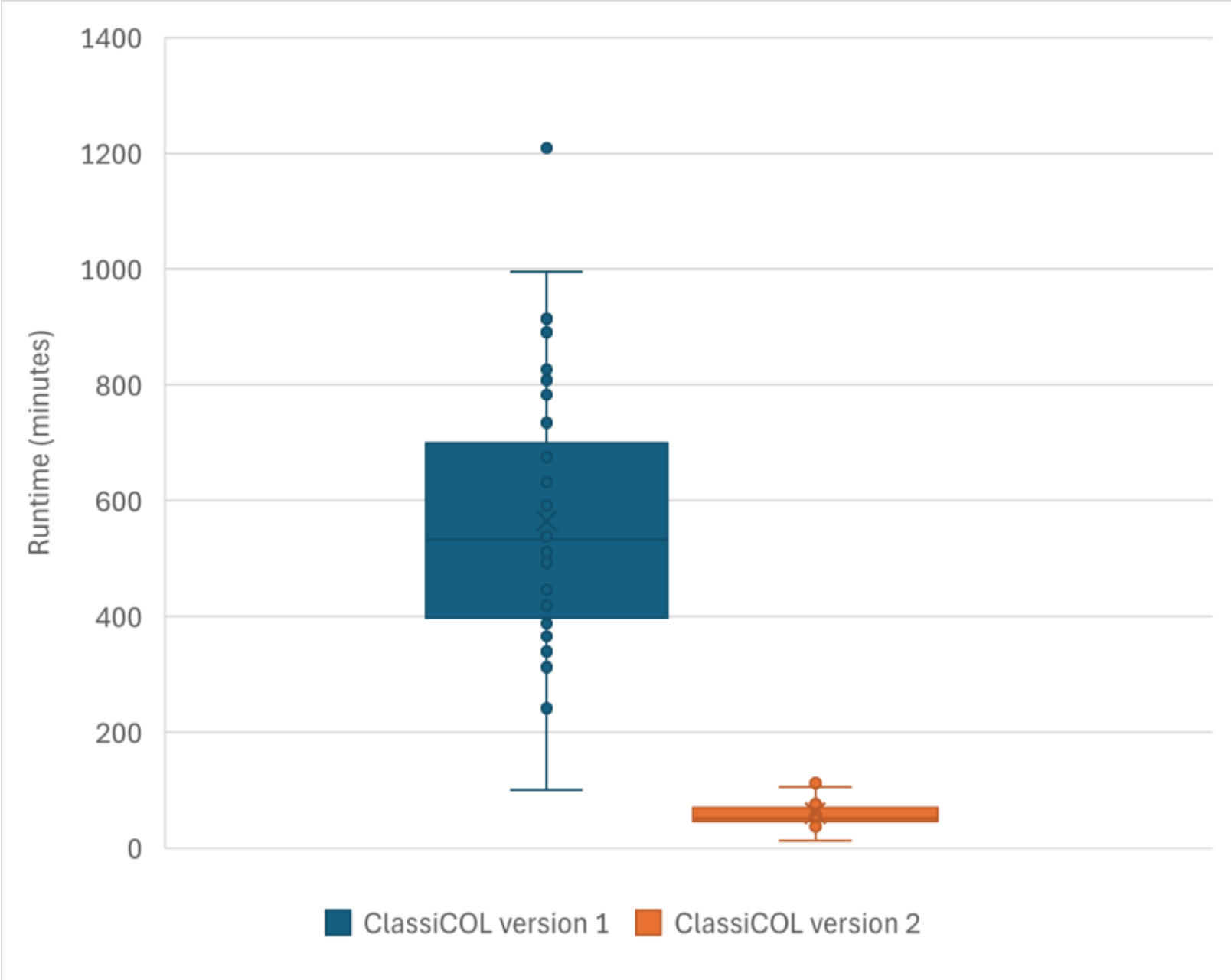

Figure S1: Computational time requirement of ClassiCOL2 compared to the original version. 42 samples with abundant collagen peptide content were analysed with the original ClassiCOL pipeline and again with the ClassiCOL2 pipeline. Time from submission to final file generation are plotted in minutes. All samples were analysed in a single batch submission. The plot shows an average 10 time reduction in computational time when using the newest version.

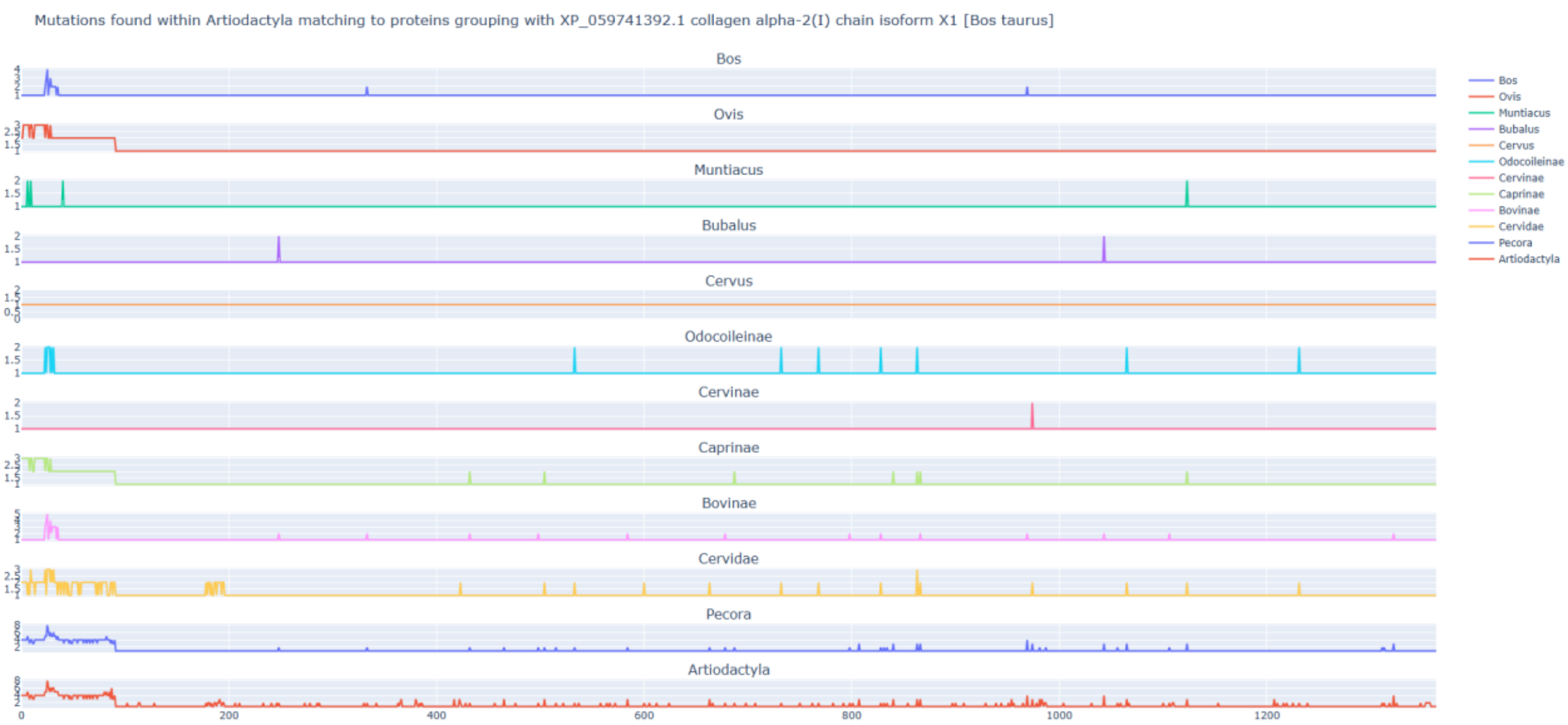

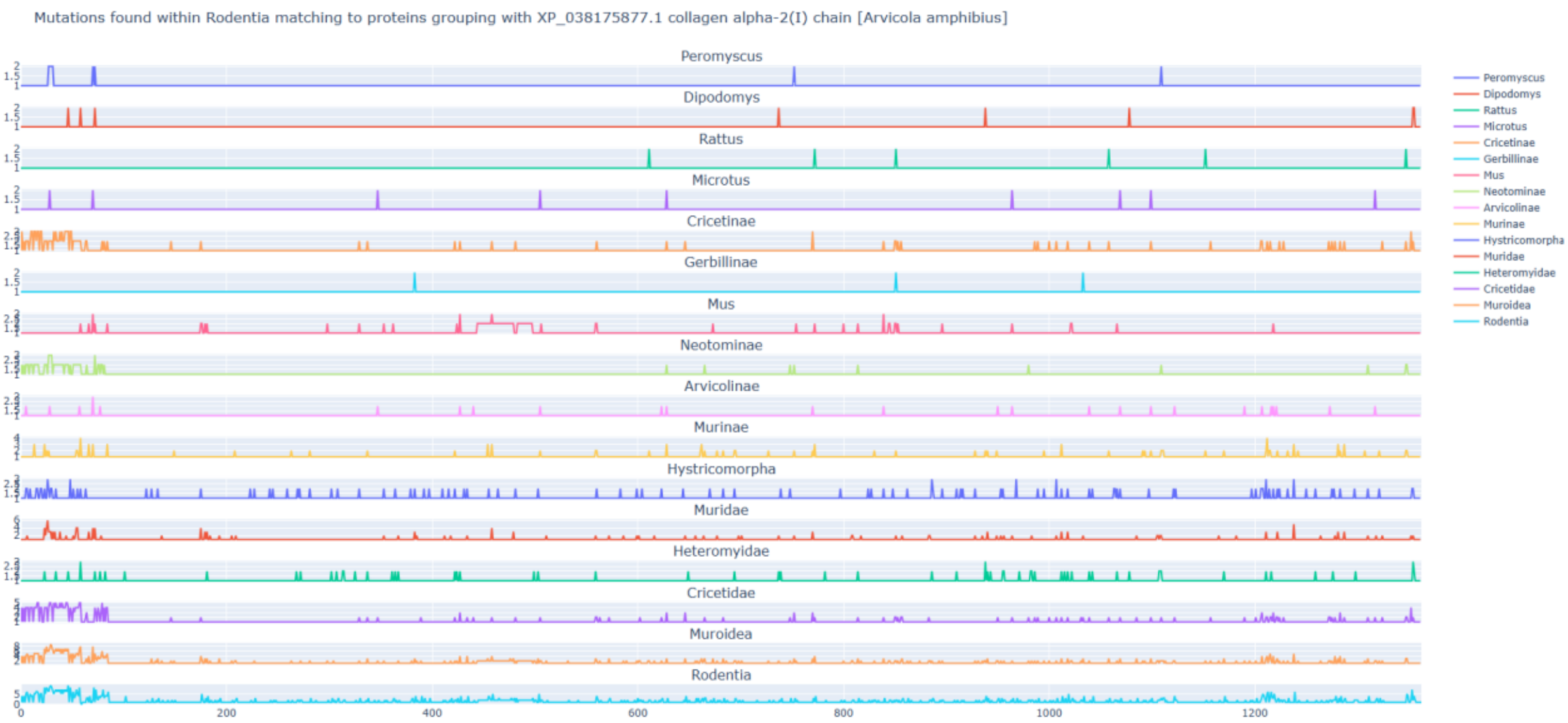

Figure S2: There is an increase in mutational space when the generational turnover increases. Plots shows the mutational space for the Laurasiatheria (top) and Rodentia taxonomic level (bottom). An increase in mutational residue location is visible comparing Rodentia to the Laurasiatheria.

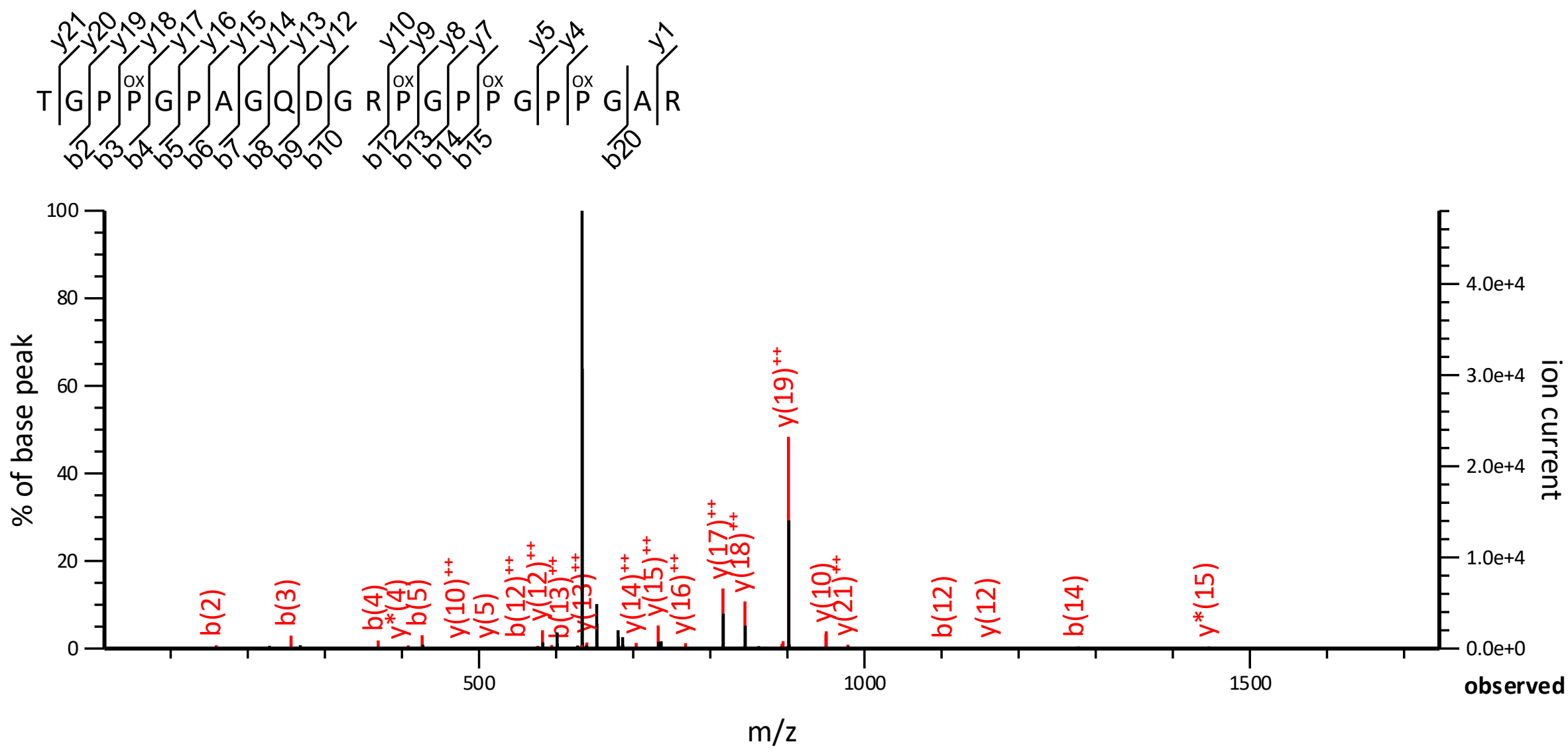

Figure S3: Peptide spectrum match of *Loxodonta africana* sample with filled out 'X'. The peptide spectrum match was found in the ED5 sample by use of Mascot. The original peptide as found in its protein sequence is TGPPGPAXQDGRPGPPGPPGAR. This spectrum thus shows the capability of search engines to account for missing amino acid residues together with the ion series coverage of the residue y15 ion and b8 ion.

Supplementary formulae

Formula S1 depicts this summarized residue score. The summarized residue score is based on the uniqueness of the residue in the database and the theoretical recentness of the mutation. In other words the overall recentness of the mutation in relation to the taxa under investigation and the uniqueness in relation to the taxa that are being compared. This means that we check the chance that the residue is unique at each taxonomic level towards the LCA of AB. Very similar to P_unique but with the difference that it sums the chance at each taxonomic node. The residue score is thus the discriminatory score of the residue when comparing taxa A and B. Taking into account that the unique mutation might be shared amongst others in the same lineage.

$$P_{recent}(A|B) = \sum_{T \rightarrow [AB, lca[} \left(1 - \frac{\sum species\ in\ DB\ under\ T\ with\ same\ aminoacid\ residue}{\sum species\ in\ database\ related\ to\ T}\right)) * (1 - (\frac{\sum species\ missing\ under\ T}{\sum all\ species\ known\ under\ T})$$

$$P_{unique}(A|B) = 1 - \left(\frac{\sum species\ in\ DB\ with\ same\ aminoacid\ residue}{\sum species\ in\ database\ related\ to\ LCA_{AB}}\right) + (1 - (\frac{\sum species\ missing\ under\ LCA_{AB}}{\sum all\ species\ known\ under\ LCA_{AB}})$$

$$Residue_{score} = P_{unique} + (\frac{P_{recent}}{distance\ to\ LCA})$$

$$Summarized\ residue\ score\ per\ taxon_{n \rightarrow LCA} = \frac{\sum residue\ score\ taxon_n}{\sum residue\ score\ ALL}$$
